# How Does Comparison With Artificial Intelligence Shed Light on the Way Clinicians Reason? A Cross-Talk Perspective

**DOI:** 10.3389/fpsyt.2022.926286

**Published:** 2022-06-09

**Authors:** Vincent P. Martin, Jean-Luc Rouas, Pierre Philip, Pierre Fourneret, Jean-Arthur Micoulaud-Franchi, Christophe Gauld

**Affiliations:** ^1^Université de Bordeaux, CNRS, Bordeaux INP, LaBRI, UMR5800, Talence, France; ^2^Université de Bordeaux, CNRS, SANPSY, UMR6033, CHU de Bordeaux, Bordeaux, France; ^3^University Sleep Clinic, Services of Functional Exploration of the Nervous System, University Hospital of Bordeaux, Bordeaux, France; ^4^Department of Child Psychiatry, Hospices Civils de Lyon, Lyon, France; ^5^IHPST, CNRS UMR 8590, Sorbonne University, Paris, France

**Keywords:** clinical decision, artificial intelligence, machine learning, clinical practice, cross-talk

## Abstract

In order to create a dynamic for the psychiatry of the future, bringing together digital technology and clinical practice, we propose in this paper a cross-teaching translational roadmap comparing clinical reasoning with computational reasoning. Based on the relevant literature on clinical ways of thinking, we differentiate the process of clinical judgment into four main stages: collection of variables, theoretical background, construction of the model, and use of the model. We detail, for each step, parallels between: i) clinical reasoning; ii) the ML engineer methodology to build a ML model; iii) and the ML model itself. Such analysis supports the understanding of the empirical practice of each of the disciplines (psychiatry and ML engineering). Thus, ML does not only bring methods to the clinician, but also supports educational issues for clinical practice. Psychiatry can rely on developments in ML reasoning to shed light on its own practice in a clever way. In return, this analysis highlights the importance of subjectivity of the ML engineers and their methodologies.

## 1. Introduction

Todays gospel in clinical psychiatry is that the field struggles with the absence of useful biomarkers, requires relying on an operationalized phenomenological level (1) and that interrater reliability for common psychiatric disorders should be strengthened by various measures of transdiagnostic symptoms (2, 3). Facing the inefficiency of the responses provided in recent decades (4), the focus has shifted to better definitions of phenotypes (5): how to refine clinical phenomenology in order to find biomarkers, improve reliability, or better qualitatively measure symptoms?

One answer to this thorny question is a refinement of the analysis of clinical judgment and reasoning, which relies on a large literature in psychiatry that is continuously developed for more than 50 years (6). While this issue is not specific to psychiatry, this domain could be chosen as a privileged clinical field to study it, inasmuch as a complex medical discipline possessing a set of deep reflections, salient questions and subtle counter-examples (7).

On one side, clinicians can be considered as a non-explicit black-box model. They make choices based on clinical decision-making processes that are not necessarily explicit. These choices are made according to an “embodied model,” i.e., a clinical model used to support the clinical reasoning and clinical decision.

In parallel, the growing interest in computational sciences allows considering “objective” automated methods, such as Artificial Intelligence (AI), and more specifically Machine Learning (ML) (8, 9). However, computational decision-making processes are created and designed by an engineer, who makes choices about the conception of such models that are not devoid of any subjectivity (10). In this way, ML engineers are driven by their experiential and subjective way of thinking, i.e., “embodied models”—like clinicians.

In this article, we hypothesize that the co-lighting of reciprocal reasoning and cross-teaching between IA and clinician reasoning could be fruitful (11, 12). This interdisciplinary dialog would not only shed light on clinical practice but also create a dynamic for the challenges of the psychiatry of the future, bringing together digital technology and clinical practice (13–15).

Thus, we do not propose to list the different computational methods designed to replace the judgment of the clinician. Rather, we aim to discuss in a systematic way how comparing clinical reasoning with data modelization could help to understand the formulation of the clinical judgment.

**Figure 1:**
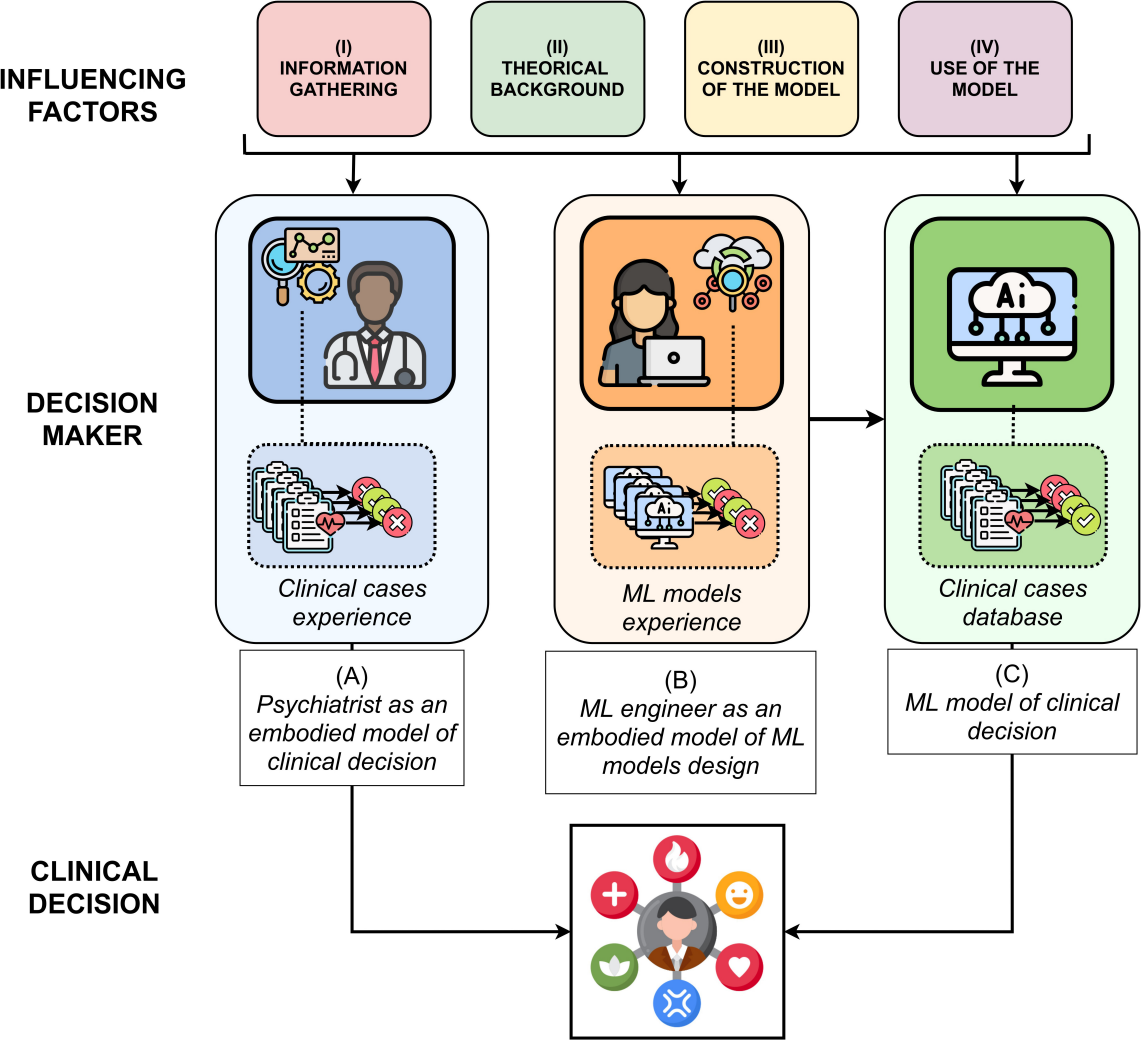
A translational roadmap in four steps accounting computational reasoning (i.e., ML engineer as an embodied model) by analogy with clinical reasoning (i.e., psychiatrist as an embodied model), in order to support clinical decisions.

The aim of this paper is to provide a *translational roadmap* comparing clinical reasoning with computational reasoning, as proposed in Figure 1. Such a translational roadmap corresponds to a set of definitions and tools, necessarily not exhaustive, which allows feeding the understanding of clinical reasoning for the ML engineer and the understanding of ML for the clinician. Consequently, this article is intended for both psychiatrists interested in ML and ML engineers interested in clinical reasoning.

First, based on the relevant literature on clinical ways of thinking (16–25) and ML (26–32), we have differentiated the process of clinical judgment into four main stages. Such a translational effort allows to analytically detail each step of the two modes of clinical and computational reasoning. Secondly, we have brought to light parallels between clinical reasoning, the engineer methodology to build a ML model and the ML model itself. Such identification necessarily leads us to distinguish the decision-making model of clinicians, embodied with them, the decision-making model of ML engineers (about how to design a ML model), embodied with them, and the ML models themselves. The details of these parallels are decomposed in four steps: 1) collection of variables, 2) theoretical background, 3) construction of the model, 4) use of the model. They are detailed in Table 1. A more complete version of this table with examples is available in Supplementary Table S1.

**Table 1:** Analogies between clinical and machine learning decision-making processes.

**Steps**	**(A) Clinician model of clinical judgment**	**(B) ML engineers model of ML model**	**(C) ML model of clinical judgment**
I) Collection of variables Information gathering
	*a) Nature of the variable*		
	Medical records (symptoms, risk factors, harms, …)	Uni- or Multimodal inputs : chosen by the designer	Extracted features (except for end-to-end models)
	*b) Labeling of the variables*		
	Naming	Choosing computer variable names when coding	-
	*c) Prioritization of the variables*		
	Choice of the most central variables in the diagnosis	Algorithm/criteria that will prioritize the variables	Feature selection or clustering algorithms
	* d) Finesse of the variables*		
	Dynamic refinement of the clinical interview according to her/his expectations	Multimodal, multitemporal and multidimensional datasets	Multimodal, multitemporal and multidimensional models
	* e) Relations between variables (causality)*		
	Understanding of symptoms in mutual interaction	Still the domain of the engineer	Not (yet) existing
	* f) Categorization of the patient*		
	Projection on a profile or a group of typical profiles	Categorization of the data / of the label	Projection on representative dimensions (features)
II) Theoretical background
	* a) Models of psychiatry*		
	Medical, Biopsychosocial, neurobiological	Trends in ML models	-
	* b) EBP*		
	Guidelines and literature	Guidelines and trends	-
	* c) Personalities*		
	The clinicians values	Personality of the engineer	-
III) Construction of the model
	* a) Training of the initial model*		
	Psychiatric pedagogical training	Computer Sciences classes	Training with best hyperparameters
	* b) Experience and expertise*		
	Job tenure and extent of knowledge on a domain	Job tenure and extent of knowledge on a domain	Changes of the model with new samples (MLops)
	* c) Cognitive reasoning*		
	Theory- or data-driven	Theory- or data-driven	Degrees of liberty
IV) Use of the model	* a) Uncontrollable factors*		
	Clinician salary	Engineer salary	Hardware cost
	Time		
	Patient's compliance, tolerance, adherence	Tolerance and adherence of the patients but also of the clinicians	-
	* b) External influences*		
	Team, social and institutional pressures and requirements	Team, social and institutional pressures and requirements	-
	Interdisciplinarity		Transfer Learning

*ML: Machine Learning*.

## 2. Discussion

In each of the four steps, we detail the factors taking part in (A) the embodied clinical model of the psychiatrist; (B) then the embodied model of the engineer ML; (C) then if it exists, the ML algorithm which allows rendering the clinical decision.

### 2.1. Variables and Information Gathering

The first step of clinical reasoning is data gathering, divided into two main steps: collection of variables, including the choice, labeling, prioritization, and granularity of these variables; and relations between them. The collection of variables is a sensible step, during which the characteristics of the patients are projected into main dimensions.

#### 2.1.1. Collection of Variables

##### 2.1.1.1. Nature of the Variables

First, numerous variables can be collected both by the clinicians and the ML engineers. Regarding the first, these variables can be symptoms, risk factors, harms and values, external validators, or even biomarkers (33).

On their side, data scientists in charge of the database design also select one or multiple input data that can be measured, e.g., voice, facial expressions, Internet of Things (IoT) data, biological, or genetic data (34).

##### 2.1.1.2. Labeling of Variables

Then, clinicians annotate the phenomena expressed by their patients, to create data potentially integrated into their clinical model, with a potential loss of information (16, 19).

In a smaller amount, the same importance of labellization occurs when the ML engineer names a variable or uses an alias for a parametrized and complicated function (35, 36).

##### 2.1.1.3. Prioritization of the Variables

These variables are hierarchically selected from the patient according to their expected importance, i.e., their epistemic gain, with a view to prediction and/or prognosis and/or clinical care. For instance, psychiatric medication is generally prescribed in a transdiagnostic manner, based on symptoms belonging to different diagnostic categories (37). Indeed, the same treatment for acoustico-verbal hallucinations can be given, whether it is a diagnosis of schizophrenia or another type of delirium (e.g., paranoid).

On their side, to prioritize the variables, ML engineers choose an algorithm that corresponds to the criterion they want to satisfy, e.g., according to their discriminative power (38).

##### 2.1.1.4. Finesse of the Variables

The clinician adjusts the finesse of the collected variables through the clinical interview (39). For instance, if the patient describes a "sleep disorder," she/he will seek to question the type of disorder, e.g., insomnia, and more specifically early or late insomnia (40).

On the other side, the available finesse both in terms of time and concept for ML algorithms are restricted to (static) datasets, chosen by the dataset designer (41).

#### 2.1.2. Relations Between Variables (Causality)

Clinicians account for a causal chain between symptoms, i.e., correlations between variables (42, 43). Clinicians tend to think intuitively about psychiatric disorders in terms of a mutual causal influence between clinical manifestations (44).

Regarding ML, the transition from statistical models to causal learning is one of the biggest challenges for AI in the coming years: causal AI is not (yet) a reality. Indeed, the concern of causality is expressed in the computer science community through explainable AI, which aims at extracting clues from black-box models in order to allow engineers to interpret them and make themselves the causal chain between the inputs of the system and the label (45).

However, recent and current works are focusing on causal learning, i.e., the design of models that “contains the mechanisms giving rise to the observed statistical dependences” (46) which is one of the biggest challenges of ML for the next few years.

### 2.2. Theoretical Background

Once the data is collected, different theoretical backgrounds (i.e., set of rules) allow structuring the data.

#### 2.2.1. Models of Psychiatry

The embodied models of clinicians are rooted in different theoretical traditions. For example, the medical model corresponds to a vision primarily guided by the consideration of common causes allowing explaining a set of symptoms, sometimes anchored in a mainly neurobiological model. These different theoretical traditions influence the definition of what a psychiatric disorder is [e.g., harm and dysfunction in the Wakefield model (47), which postulates that a medical disorder is defined by a dysfunction resulting in harm to the patient], and by extension the consideration of the patient. Thus, psychiatrists are regulated by theoretical sets of laws and rules structuring their way of conceiving their embodied clinical model (and therefore of structuring their data).

This consideration echoes the different trends existing in the machine learning field which is at the crossroad of multiple different approaches. One example is the recent advent of data-driven AI led by Andrew Ng, in opposition to the classical model-driven approach that is usually employed in ML (48). In the latter, the focus is on the design of the machine learning model (e.g., a classifier or regressor) that will model the best relationship between the features extracted from this data and the label on a given dataset, whereas data-driven AI focuses on data engineering processes to obtain higher performances with a given model.

#### 2.2.2. Evidence-Based Practice

Psychiatry is regulated by a set of guidelines and recommendations which are beyond the control and subjectivity of the clinician. Some of these guidelines are internationally recognized, e.g., the National Institute for Health and Care Excellence (NICE) Guidelines or the American Psychiatric Association Practice Guidelines.

Although there are some guidelines on the application of ML in the medical field [e.g., (49)], guidelines on how to build such systems are few and rarely used or followed. Usually, machine learning engineers follow implicit guidelines picked from “rules of good practice” and some reference conferences, articles or ML engineers that make “jurisprudence” and serve as guides for machine learning engineers to design their models [e.g., the yearly conference NEURIPS (50), exposing the latest trends in artificial intelligence].

#### 2.2.3. Personalities

Clinicians' personality will influence their theoretical choices. For instance, if they are inclined to take risks, or conversely to be rather conservative, they will tend to favor different diagnostic practices. Likewise, uncertain decisions will lead to different diagnostic, therapeutic responses and more generally different decision-making paths depending on the clinicians and patients willingness to take risks.

Regarding ML engineers, personality also influences their choices. Indeed, depending on their recklessness (or adventurousness) or conservatism, their sense of the aesthetics, and other aspects of their personality, engineers will choose one implementation over another (51, 52). For example, enterprising engineers try new and funky pipelines despite the risk of low performances, while “conservative” engineers stick to state-of-the-art-inspired pipelines (53).

### 2.3. Construction of the Model

After having collected a set of data under the influence of the theoretical background, the structuring of this data is done by the clinician or ML engineer / ML algorithm according to the training of the model, experience and expertise, and cognitive reasoning.

#### 2.3.1. Training of the Initial Model

Educational training in psychiatry is largely based on case studies. This repetition of confrontations with clinical cases (fictitious or not) will allow clinicians to be trained. They will be rewarded or penalized according to their skills, which will allow their internal model to be trained in front of new cases (e.g., the National Classifying Exam in France or the Psychiatry Certification examination in the US).

Similarly, at the end of their schooling, the ML engineers models of problem solving with ML has been shaped by the examples they have dealt with in class, whether they are fictional examples or real data.

Regarding the ML model, an initial version is trained with the whole dataset (i.e., input variables annotated with some clinical label) when the best pipeline and configuration (or “hyperparameters”) have been selected, before being deployed in real conditions and confronted to new data (54). Thus, the training of an initial model is undertaken systematically.

#### 2.3.2. Experience and Expertise

Learning is not a static process. The models of both psychiatrists and engineers do not stay in their initial state. The constitution of the theoretical background is largely influenced by two factors: time and expertise.

Concerning time, the number of cases encountered by the clinician changes their initial theoretical background. For example, young clinicians tend to follow their personal readings and training will open up to other theoretical backgrounds over time (55). Regarding expertise, the knowledge of clinicians in one area influences their practices in other areas (i.e., diagnostic and therapeutic practice). For example, clinicians working with neuroscientists in a specialized center for autism might tend to apply their neurodevelopmental models to other psychiatric disorders (16).

The same tenet about time and expertise factors apply to ML engineers. On one hand, the more they confront diversified problems and interact with their peers, the more their perspective of the field grows and allows them to embrace new theoretical backgrounds (10). On the other hand, engineers working in a neurocomputational or in a theoretical informatics team do not embrace similar problems in the same way, and develop different approaches to solve them.

Regarding the ML model, when the pipeline is put into production (real-life situation), no background modification is possible (the pipeline is static—it has been chosen during the training phase), but a shift of specialization is possible depending on the data it is fed with (54, 56).

#### 2.3.3. Cognitive Reasoning

Clinical cognitive reasoning is a field of research in its own right. In this article, we have specified how the clinical decision pertains to each of these steps. However, the way of using the embodied clinical model also has parallels with the embodied model of the engineer ML and the ML algorithm.

Indeed, clinicians use each set of variables for each patient, i.e., they build as many models as there are cases, based on their theoretical rules. Thus, clinicians can rely more or less on theoretical hypotheses or on data presented to them in the clinical practice, in order to build their model. Consequently, they can either strongly constrain their data with predefined laws in a theory-driven manner, or on the contrary use clinical reasoning by trial and error in a data-driven manner (e.g., constraining their model with descriptive categorical criteriological approaches).

On the contrary, ML engineers always try to balance their theoretical knowledge with regards to the data. Indeed, significant parts of the model of the engineer rely on rules that depend on data distributions and tasks (26, 32).

Regarding ML models, when they are put into production, the only parameter that could be set regarding their degree of freedom is the choice to fine train the model with the new samples, and if so, the importance to give to them (54, 56).

### 2.4. Use of the Model

#### 2.4.1. Uncontrollable Factors

Considering diagnostic and therapeutic issues constitute only part of the medical care. Indeed, other challenges, such as the cost of a clinician to society, necessarily influence clinical practice. For instance, on a day of hospital consultation, clinicians should have seen a certain number of patients in consultation, requiring that they limit the time dedicated to each of them. Thus, cost and time are two intrinsically related uncontrollable factors that should be considered in the clinical modeling of practitioners.

The same factors shape the engineers work, but through another temporality: these factors influence the ML engineer and ML system during the design of the ML model, not during its use. In fact, both the engineers salary and the number of projects they are working on may affect the way they code. For instance, engineers could limit themselves to pre-coded pipelines [such as the Python library Sci-kit learn (57)] because of lack of time, whereas some solutions could have worked better but would have required more time. Time and cost factors also influence the precision of the parameters of the ML model, e.g., through the material needed to accelerate their computation (e.g., GPU cost).

#### 2.4.2. External Influences

To these constraints are added external influences, such as clinicians team within which they work, and more generally the social and institutional pressures and requirements that weigh on them. For instance, these pressures and requirements are more or less burdensome depending on novice decision makers or experienced clinicians—the latter theoretically resisting conflicts and external constraints. Finally, beyond the external constraints, clinicians work voluntarily in an interdisciplinary manner. Such an issue requires compromises on the part of clinicians, who find themselves at the intersection of external viewpoints and which modifies their clinical judgment.

The same constraints can be applied to ML engineers, depending on the specialization they come from and the environment they are working in.

An equivalent of interdisciplinarity for ML models could be transfer learning, in which a model trained on a specific model from one domain is fine-trained on new data and used in another task (58).

## 3. Conclusion

From this fruitful comparison emerges the idea that the understanding of AI does not only bring methods to the clinician, but also sustains secondary benefits: due to the necessary decomposition of its operation (59), it supports educational issues for clinical practice. Thus, understanding how the ML works could inform clinical reasoning. Far from the possibility that psychiatric diagnosis no longer requires clinicians (60), the discipline can conversely rely on developments in ML reasoning to shed light on its own practice in a clever way.

In return, the understanding of such an embodied clinical model could help to understand the importance of the subjectivity of ML engineers. While this effect has already been documented in the literature [e.g., (10)], this comparison brought into light some of the factors involved in the choice of engineers when designing a ML model, continuing the deconstruction of the myth of a desubjectivized and neutral AI.

Such developments proposed in this article not only have an interdisciplinary scope, allowing for the understanding of the empirical practice of each of the disciplines (psychiatry and ML engineering), but also a transdisciplinary scope of clinical reasoning, providing new methods for dialectically grasping what cannot be understood through the prism of a single discipline—even in interaction with another field.

This three-model dialog (embodied model of the psychiatrist, embodied model of the engineer and ML model) only partially considers, however, a crucial fourth actor: the embodied model of subjectivity of the patients. Like those presented in this perspective paper, the latter collects information through a theoretical background which is then processed with its own embodied model. Just as the comparison between clinical reasoning in psychiatry and reasoning in ML has led to the emergence of new methods for studying clinical decision-making, a dialog between these fields and the patients subjectivity would complement this transdisciplinary approach, while placing patient ethics at the center of the discussion.

## Data Availability Statement

The original contributions presented in the study are included in the article/Supplementary Material, further inquiries can be directed to the corresponding author.

## Author Contributions

VM: writing, original draft preparation, conceptualization, and editing. CG: writing, original draft preparation, conceptualization, methodology, and editing. J-LR: supervision, reviewing, and validation. PP: reviewing, methodology, and validation. PF: reviewing, resources, and validation. J-AM-F: supervision, methodology, reviewing, resources, and validation. All authors contributed to the article and approved the submitted version.

## Conflict of Interest

The authors declare that the research was conducted in the absence of any commercial or financial relationships that could be construed as a potential conflict ofinterest.

## Publisher's Note

All claims expressed in this article are solely those of the authors and do not necessarily represent those of their affiliated organizations, or those of the publisher, the editors and the reviewers. Any product that may be evaluated in this article, or claim that may be made by its manufacturer, is not guaranteed or endorsed by the publisher.
