# Supplementary material for: How Does Comparison With Artificial Intelligence Shed Light on the Way Clinicians Reason? A Cross-Talk Perspective
Source: Front Psychiatry. 2022 Jun 9;13:926286. doi: 10.3389/fpsyt.2022.926286 (PMC9218339; doi:10.3389/fpsyt.2022.926286)
Supplement: Supplementary file 1 [file Table_1.DOCX]

Supplementary Material

**Table S1**. Analogies between clinical and machine learning heuristics.

ML: Machine Learning

| **Steps** | **Clinician Model of clinical judgment** | **ML engineer’s model of ML model** | **ML model of clinical judgment** |
| --- | --- | --- | --- |
| (I)  Collection of variables / Information gathering | 1. Nature of the variable | | |
|  | Medical records (symptoms, risk factors, harms, biomarkers) | Uni- or Multimodal inputs: chosen by the database designer | Extracted features (except for end-to-end models) |
|  | *Ex: Listening to the patient’s speech, looking his/her posture, facial expression* | *Ex: Speech, eye tracking, smartwatch data* | *Ex: Voice frequency, eye perclos, number of steps/day* |
|  | 1. Labeling of variables | | |
|  | Naming | Choosing computer variable names when coding | *-* |
|  | *Ex: What appears in the medical record is not the phenomenological variable as experienced by the patient, but just its name, leading to a loss of information by projection of reality on the closest name.* | *Ex: kmean = sklearn.cluster.KMeans(n_clusters=8, *, init='k-means++', n_init=10, max_iter=300, tol=0.0001, verbose=0, random_state=None, copy_x=True, algorithm='auto')* |  |
|  | 1. Prioritization of the variables | | |
|  | Choice of the most central variables in the diagnosis | The engineer chooses the algorithm/ the criteria that will prioritize the variables | Feature selection or clustering algorithms |
|  | *Ex: Anhedonia and sad mood (monothetic criteria) in major depressive disorder; risk of hyponarrativity.* |  | *Ex: Principal Components Analysis, SelectKBest* |
|  | 1. Granularity of the variables | | |
|  | Dynamic refinement of the clinical interview according to her/his expectations (clinical epistemic gain) | Multimodal, multitemporal and multidimensional datasets | Multimodal, multitemporal and multidimensional models |
|  | *Ex: “Insomnia” for the major depressive episode versus three types of insomnia (initiating, maintaining, early) as part of a consultation specializing in sleep medicine* | *Ex:* [MSLTc](https://www.frontiersin.org/articles/10.3389/fdgth.2021.686068/full) | (coming soon) |
|  | 1. Relations between variables (causality) | | |
|  | Understanding of symptoms in mutual interaction. | Still the domain of the engineer | Not (yet) existing |
|  | *Ex: Traumatic event > Anxiety > Insomnia > Irritability* | *Ex: Efforts invested in XAI* | *Ex: Adversarial attacks* |
|  | 1. Categorization of the patient | | |
|  | Projection on a profile or a group of typical profiles | Categorization of the data/labels | Projection on representative dimensions (features) |
|  | *Ex: Risk of reification* | *Ex: “Depressive people vs. Non-Depressive”* | *Ex: Bag-of-Words* |
| (II)  Theoretical background | 1. Models of psychiatry | | |
|  | Medical, Biopsychosocial, neurobiological | Trends in ML models | - |
|  | *Ex: Psychiatric disorder is defined as a harmful dysfunction* | *Ex: Data-driven AI vs. model-driven AI* |  |
|  | 1. EBP (guidelines) | | |
|  | Guidelines and literature | Guidelines and trends | - |
|  | *Ex: National Institute for Health and Care Excellence Guidelines or American Psychiatric Association Practice Guidelines* | *Ex: NEURIPS* |  |
|  | 1. Personalities | | |
|  | The clinician’s values and non-medical factors influence the theoretical choice | Personality of the engineer | - |
|  | *Ex: Uncertain decisions will be different depending on the clinician’s and patient’s willingness to take risks or be conservatist (e.g., to announce the diagnosis).* | *Ex: Funky pipelines or reproduction of the state of the art* |  |
| (III)  Construction of the model | 1. Training (of the initial model) | | |
|  | Psychiatric pedagogical training | Computer Sciences classes | Training on the whole dataset with the best hyperparameters  (First step of Machine Learning operations*)* |
|  | *Ex: National Classifying Exam (France) or Psychiatry Certification examination (US)* | *Ex: CS classes, online workshops* |  |
|  | 1. Experience and expertise | | |
|  | Job tenure (number of cases) and extent of knowledge on a domain (specialization) | The experience of the engineer can make him/her choose one strategy or another. | Changes of the model with new samples (MLops) |
|  | *Ex: More details will be gathered and more associations between items will be made if the clinician is experienced and / or more specialized.* | *Ex: engineers working in a neurocomputational or in a theoretical informatics team do not embrace the problems in the same way* | *Ex: shift of specialization when fed with new data* |
|  | 1. Cognitive reasoning | | |
|  | Theory- or data-driven (constraints) | Theory- or data-driven (constraints) | Keep the initial model or adapt to new data |
|  | *Ex: More or less hypothesis testing versus clinical reasoning by trial and error* | *Ex: Choice of the model depending on data* | *Ex: Fine-training with new samples* |
| (IV)  Use of the model | 1. Uncontrollable factors | | |
|  | Cost: clinician salary | Engineer salary | Hardware cost (training) |
|  | *Ex:* [$260k](https://www.indeed.com/career/psychiatrist/salaries) | *Ex:* [$95k-250k in the United States](https://datasciencedegree.wisconsin.edu/data-science/data-scientist-salary/) | *Ex: GPU + maintenance costs* |
|  | Time | Time | Hyperparameters research plane (training) |
|  | *Ex: The clinician has a limited number of possible consultations per day.* | *Ex: The engineers could limit themselves to pre-coded libraries that are not optimal for his/her problem* | *Ex: ([Claesen 2015](https://arxiv.org/abs/1502.02127))* |
|  | Patient’s compliance, tolerance, adherence | Tolerance and adherence of the patients but also of the clinicians |  |
|  | *Ex: The patient’s value system may not match the clinician’s medical model.* | *Ex:* [(Bourla 2018)](https://www.ncbi.nlm.nih.gov/pmc/articles/PMC6315247/) |  |
|  | 1. External influences | | |
|  | Team, social and institutional pressures and requirements on the clinician | Team, social and institutional pressures and requirements on the engineers |  |
|  | *Ex: Conflicts or novice decision makers* | *Ex: Conflicts or novice decision makers* |  |
|  | Interdisciplinarity | Interdisciplinarity | Transfer learning from another task |
|  | *Ex: Intersection of external viewpoints modify clinical judgment* | *Ex: Engineer from Bioinformatics or Neurosciences* | *Ex: (*[*Wang et al 2019*](https://www.frontiersin.org/articles/10.3389/fpsyt.2019.00205/full)*)* |
